# Modified glatzel mirror test reproducibility in the evaluation of nasal patency

**DOI:** 10.1016/S1808-8694(15)31091-0

**Published:** 2015-10-19

**Authors:** Silvana Brescovici, Renato Roithmann

**Affiliations:** 1 Master's degree in internal medicine. Speech therapy professor, Universidade Luterana do Brasil (Brazilian Lutheran University); 2 Post-doctorate in otorhinolaryngology, Adelaide University. Adjunct professor of otorhinolaryngology and head & neck surgery, Universidade Luterana do Brasil (Brazilian Lutheran University). Adjunct professor of anatomy, Universidade Federal do Rio Grande do Sul (Rio Grande do Sul Federal University). Universidade Luterana do Brasil (Brazilian Lutheran University) and Universidade Federal do Rio Grande do Sul (Rio Grande do Sul Federal University)

**Keywords:** glatzel mirror, nasal obstruction, nasal patency

## Abstract

The Glatzel Mirror (GM) is used to evaluate nasal patency. Validation studies are not available in the literature. This paper aims to verify the GM test reproducibility and the correlation between the intra-subject condensation area and nasal patency subjective perception.

**Methods:**

This is a prospective study. 25 subjects were evaluated with the GM for five consecutive minutes, every half an hour for 4 hours; every day, beginning in the early afternoon, every Thursday for five consecutive weeks. A visual analogue scale was used to evaluate nasal patency perception in all periods.

**Results:**

The total correlation coefficient (right + left areas) found between the condensation area and the subjective perception was r = 0.04 (p = 0.37). On the left side it was r = 0.08 (p = 0.09) and on the right side r = 0.05 (p = 0.28). The mean unilateral variation coefficient was less than 15% and the total was less than 12%, regardless of the time period interval between test and re-test.

**Conclusion:**

We did not observe any significant correlation between the subjective perception of breathing and the condensation area. Unilateral variability was higher than the total (right + left area) and the test variability was the same between the different time periods of measurements.

## INTRODUCTION

Use of the Glatzel mirror (GM) is an old and simple technique for objectively and momentarily verifying the nasal patency. Few otorhinolaryngologists still use this tool, although many speech therapists recommend it for assessing and monitoring mouth breathers.^1^, ^2^, ^3^, ^4^

Zwaardemarker^5^, ^6^ first described the technique, which Glatzel^6^, ^7^ later popularized; it has been in use for over 100 years.^5^ This method for objectively evaluating nasal respiratory function consists of observing the condensation of exhaled air on a cold metal surface. The clinical examiner may obtain a momentary assessment of nasal patency by comparing the condensation area of each nasal fossa. This technique has helped understand nasal cycles,^5^, ^6^^,^^8^ and has been used in monitoring patients after nasal surgery.^9^, ^10^ There have been, however, few studies validating the data obtained by using the GM.^6^^,^^11^

Objective tests of nasal function should ideally be comfortable for patients, accurate, standardizable, easily done, clinically applicable, and should not affect the nasal anatomy and physiology.^12^ Such tests should also be reproducible, which is the possibility of producing consistent results when repeated independently.

Rhinomanometry and acoustic rhinometry are the tests that are usually employed in nasal physiology research centers. The former measures transnasal airflow and the latter calculates the complete intranasal cross-sectional area.^13^ These tests partially fulfill the abovementioned criteria, but require sophisticated devices. Acoustic rhinometry is a static test that does not require subjects to actively breathe during testing; it appears to show less test retest measurement variation.^7^^,^^14^, ^15^ Rhinomanometry measures transnasal air flow while the subject breathes nasally; it shows more variation between measurements.^15^, ^16^, ^17^

Verifying the reproducibility of GM measurements at different times might support improved data interpretation, and therefore its test retest value. Furthermore, investigating the correlation between subjective perception of nasal patency and mirror measurements may provide a theoretical basis for the clinical use of this tool, which would be desirable. And finally, the GM could become a valuable tool for the initial screening of nasal obstruction and predominantly mouth breathing. The purpose of this article was to investigate the correlation within subjects between the subjective perception of nasal patency and the objective measurements obtained by the GM. A second aim was to investigate the minute-by-minute variability of consecutive measurements of nasal patency as measured by the GM, and to study the behavior of nasal patency by measurements with the GM in independent time periods.

## MATERIAL AND METHOD

A cross-sectional study was carried out in employees of a legal department in a company located in the city of Porto Alegre, Rio Grande do Sul state, between April and May 2003. The sample size was 20 subjects, calculated using Gertner et al.'s^9^ parameters. To account for possible losses, 25 subjects were chosen; 14 were male and 11 were female, aged between 22 and 47 years (mean = 31 years). Subjects answered a standard questionnaire proposed by Lund,^17^ and underwent anterior rhinoscopy done by an otorhinolaryngologist.

Smokers were excluded, as were subjects that presented upper airway infection on the day of the exam or within the past 14 days. Other exclusion criteria were the presence of polyps, nasal tumors, septal perforation, prior surgery of the palate or nose, subjects using decongestionants, antihistaminic drugs, anticholinergic substances or topical/systemic corticosteroids chronically within the past three months, subjects reporting having thyroid, lung or cardiovascular diseases, and pregnant or menopausal women. All patients were asked to refrain from drinking alcoholic beverages and to inform whether any medication was used before evaluation.

The same person made all of the measurements in the same site. The temperature (22 to 24°C) and relative air humidity (50 to 65%) of the test site were kept constant throughout testing. The following protocol was applied:

1) acclimatizing - participants remained seated for 30 minutes in the test environment. During this period, the patients answered the questionnaire and the physical examination was done;
2) participants filled in a visual analog scale (VAS) on global perception and unilaterally by occluding the opposite nostril;
3) participants breathed on the GM.

The sequence of measurements was done as follows: minute by minute - five measurements each 60 seconds (s); every half hour - five measurements each 60 seconds, checked each 30 minutes during four consecutive hours; day by day - five measurements each 60 seconds, from Monday to Friday, in preset hours; week by week - five measurements each 60 seconds, on a preset day of the week during five consecutive weeks.

A 100mm length^18^ VAS was used for the subjective perception of nasal patency; zero was marked as “fully unobstructed,” and 100 was marked as “fully obstructed.,” Subjects were asked to mark a position along the VAS line that best described how they perceived their nasal patency. The distance between the end of the VAS line (fully obstructed) to the point that was marked was measured and taken as a nasal patency grade. Participants were instructed about the VAS and the evaluator was blind to each result until the end of the five minute-by-minute measurements.

**Figure 1:**
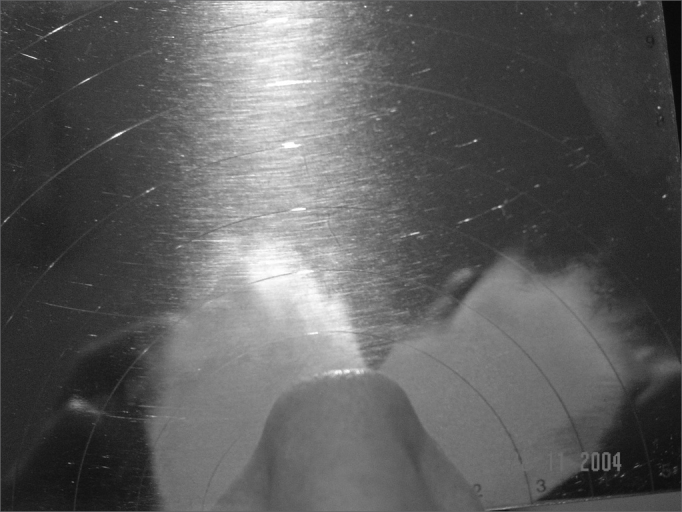
The modified Glatzel mirror.

Specially made metal plates, as described by Gertner et al.^9^ (Figure 1), were used for an objective assessment of nasal patency. This mirror was modified by placing a millimeter scale over the plate, which was not included in the mirror originally described by Glatzel; another difference was to calculate the area of the ellipse, as described later in this article. Mirror measurements were done with seated subjects and the head in the orthostatic position. The metal plate was placed horizontally under the nostrils of participants, placing the mirror's zero point under the collumela. Participants were asked to breathe slowly through both nostrils, with no inspiratory or expiratory effort, keeping the mouth and eyes closed. The first condensation was discarded and the second condensation was marked with an overhead transparency-marking pen on the mirror itself and subsequently copied by transparency to standard paper.^2^ Care was taken when handling the plate to keep it from heating.

**Figure 2:**
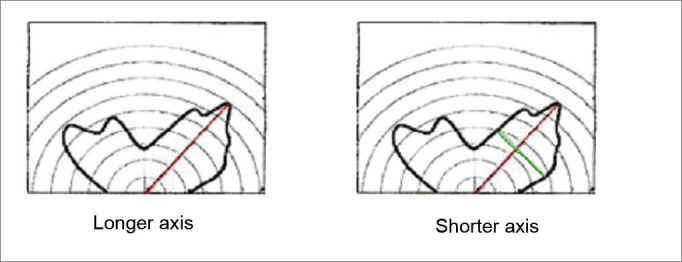
Measurement of the greater and lesser axes of a nasal condensation area.

The condensation contour was measured along is greater and lesser axes, on the left and right sides. The greater axis was obtained starting from the central point (zero mark) until the longest dimension (within the contour. The lesser axis was also selected; it was always perpendicular to the greater axis (Figure 2). The same ruler was used in all measurements (Trident triangular scalimeter - architect's scale - model ME-15/1). A second evaluator measured again the greater and lesser axes. New measurements were taken if the results did not coincide. Finally, the condensation area was calculated by using the mathematical formula for the ellipse (S=a.b.π) proposed by Gertner et al.^9^ The sum of right and left nasal fossa unilateral values was calculated for the final assessment.

Pearson's correlation coefficient was used for assessing the correlation between VAS scores and GM measurements (mean of five measurements). The analysis of variance was used for estimating residues, as this preliminary step defined intra-subject correlations.

Variation coefficient values were calculated for each of the 25 subjects to check the mirror's variability of permeability in minute-by-minute, hour-by-hour, day-by-day and week-by-week measurements. The mean of five measurements was used for hour, day and week intervals.

The SAS software, version 8.2, was used for the analysis of variance (ANOVA); the purpose of this was to check whether any variability that was encountered was defined by a “time,” effect between the measurements of each subject or if variability occurred due to differences among the 25 subjects. The significance level was 0.05.

The Research Ethics Committee of the Porto Alegre Clinical Hospital (Hospital de Clinicas de Porto Alegre), Rio Grande do Sul Federal University, approved the study; its registration number in the Research and Post-Graduation Group was 01407. All participants signed a free informed consent form before participating in the study.

## RESULTS

The correlation between the VAS and the GM scores revealed that a statistically significant positive correlation was present in only 32% of subjects. Of these, 75% occurred in unilateral measurements and 37% occurred in the total measurements (one of the subjects presented a correlation both in unilateral and in total measurements). A negative correlation was found in 16% of cases.

The correlations have no statistical significance when the results are considered jointly. The total correlation coefficient (right and left sides) was r=0.04 (p=0.3761); the left correlation coefficient was r=0.08 (p=0.0984), and the right correlation coefficient was r=0.05 (p=0.2862).

Table 1 shows the variation coefficients for minute-by-minute measurements. The total variation coefficient (left + right) is lower than the unilateral variation coefficients.

**Table 1:** Reproducibility of the nasal condensation area on the Glatzel mirror in five one minute interval measurements

	Subjects	Area	VC%	VC%	VC%
		(n)	Median	Maximum	Minimum
Left	25	475	14	42	3
Right	25	475	14	53	1
Total	25	475	11	31	2

VC = variation coefficient; n = number

Table 2 shows the variation coefficient results for four hours, five days and five weeks consecutively. Regardless of how time was expressed, analysis of variance revealed no significant intra-subject variation. In other words, variations were statistically significant only for the total area (in time expressed as hours), for the right area (in time expressed as weeks) (Table 3). Variation was significant among subjects for the right side, the left side and the total measurements. Interaction time for each subject was also statistically significant.

**Table 2:** Reproducibility of the nasal condensation area on the Glatzel mirror in independent time intervals

	Subjects	Area	Variation Coefficient (%)		
		(n)	Median (minimum-maximum)		
			Left	Right	Total
Hours	25	225	14 (3–39)	14(1–42)	11 (7–28)
Days	25	125	15 (4–38)	14(4–37)	11 (3–28)
Weeks	25	125	14 (4–39)	15(4–9)	12 (2–31)

n = number;

VC = standard deviation × 100/mean

**Table 3:** Analysis of variance taking into account time expressed as hours, days and weeks

				Left		Right		Total	
Causes of variation		df num	df den	F	P	F	P	F	P
	Time	8	176	1,21	0,2938	2,80	0,0061	3,23	0,0019
Hours	Subject	22	176	39,87	<0,0001	22,84	<0,0001	49,97	<0,0001
	Subject*Time	176	900	5,02	<0,0001	7,11	<0,0001	5,71	<0,0001
	Time	4	88	0,89	0,4708	1,08	0,3699	1,16	0,3341
Days	Subject	22	88	5,21	<0,0001	8,65	<0,0001	9,13	<0,0001
	Subject*Time	88	500	8,40	<0,0001	7,30	<0,0001	9,36	<0,0001
	Time	4	88	0,51	0,7276	2,96	0,0240	1,08	0,3721
Weeks	Subject	22	88	10,12	<0,0001	9,09	<0,0001	11,60	<0,0001
	Subject*Time	88	500	5,74	<0,0001	6,67	<0,0001	7,34	<0,0001

df num= degrees of freedom/numerator; df den = degrees of freedom/denominator

## DISCUSSION

The subjective feeling of nasal patency should ideally correlate with objective measurements of nasal function; various authors have tested this association hypothesis. Some studies have used static objective tests,^19^ while others have used dynamic tests.^20^, ^21^, ^22^, ^23^, ^24^ Still other studies have been designed for large samples,^20^, ^21^^,^^24^ others for few subjects in various situations,^23^ some in normal subjects,^23^^,^^25^ and others in subjects with nasal complaints^21^^,^^26^, ^27^ Some of the studies have demonstrated a good correlation,^21^^,^^23^^,^^27^ which has not been confirmed in other papers.^20^^,^^24^ Unilateral correlations have been better than total (bilateral) ones;^20^^,^^22^ patients appear to recognize unilateral obstruction more easily. In all of these papers, no study investigated this hypothesis by using rhinohygrometry or the GM.

Our results suggest that there is no correlation between subjective and objective measurements, which may be partly explained by the fact that the sample was composed of healthy individuals with no nasal complaints. Sipilä et al.^22^, referring to studies by Jones et al.^24^ and Naito et al.,^28^ also commented this situation. Other studies^22^^,^^24^^,^^28^ have suggested that if subjects with clear symptoms of nasal obstruction or upper nasal air resistance were included, the correlation between data might be improved. Numminen et al.^29^ reached a similar conclusion, stating that objective methods are more sensitive in recognizing changes in the nasal mucosa of subjects with nasal complaints, compared with healthy subjects.

It is possible that the causes of nasal air resistance are different from those of the feeling of airflow, since factors other than resistance affect this feeling. These factors include the thermal receptors in the vestibule and mucosa, and the mucocilliary function. Eccles et al.^30^ found that the nasal feeling of airflow, but not the resistance, is affected by inhaling menthol. Jones et al.^31^ reported a slightly increased nasal resistance, not accompanied by a corresponding feeling of obstruction, following aspirin use. The use of topical anesthetics on the nasal mucosa produces a feeling of nasal obstruction which is not accompanied by a decreased transnasal airflow.^32^^,^^33^ These observations corroborate our results, which show that there was no correlation between objective findings of the GM and the perception of permeability by subjects.

It is worth noting that, although the VAS has been widely used in rhinological research,^20^, ^21^, ^22^, ^23^, ^24^, ^25^, ^26^^,^^29^, ^30^^,^^33^ there are no studies validating this tool for assessing the subjective perception of nasal patency. Sipilä,^22^ in dividing the scale into quartiles, and Jose and Ell,^34^ in using categorical scales, found a good correlation between rhinometric measurements and subjective data. It is possible that subjective perceptions are best evaluated by means of categorical scales. We suggest, therefore, that new studies be done comparing the nasal condensation area and subjective measurements of nasal patency obtained by other methods.

On the other hand, when the correlation in each subject was investigated independently, a positive correlation was found in one third of cases, which suggests that larger condensation areas are proportional to an improved feeling of nasal airflow permeability. This may have occurred because each subject has his or her own individual scale of feeling in relation to resistance. Farley et al.^23^ found a strong correlation between the feeling and the peak inspiratory flow in a study based on repeated measurements in a small sample (five subjects). These authors suggested that each individual has a personal calibration curve, and that studies based on a small number of measurements in large samples yielded wrong estimates of subjective and objective data. Variations between individuals would be so large, that any general relation would be masked.

Furthermore, when there was a positive correlation, two-thirds had a unilateral correlation and one-third presented correlation of total variables (right and left). It is clear, therefore, that there is better correlation of unilateral values. Sipilä et al.,^22^ Panagou et al.,^35^ and Roithmann et al.^26^ found similar results. This may be because when subjects assess the total feeling or sensation, their evaluation is based on the side with increased or decreased permeability, which distorts the correlation with objective measurements.^22^

It is important to note that in some cases (16% of the current sample) the correlation was negative; in such cases, the area of condensation decreased while the feeling of permeability increased, or vice versa. These controversial clinical responses underline the need for care when interpreting single measurements of perception or objective values (resistance, condensation area, cross-sectional area, volume, peak flow and others). Thus, the nasal condensation area should not be the only parameter for measuring the perception of nasal area in patients, except when contextualized by the clinical history and the physical examination.

Reproducibility, which quantifies to which extent repeated measurements in different moments yield similar results, depends (in flow tests, such as this technique for evaluating nasal function) on three components: the features of the test instrument, the operator's technique when using it, and changes in the shape and size of airways.^15^ Care was taken to minimize any interference from these factors; data, however, is a combination of possible variations of these three components.

Care was taken to avoid heating of the metal plate when handling the test instrument and upon collecting the second expiration. However, it is not possible with this test instrument to fixate the condensation, which rapidly disappears and is therefore highly dependent on the operator. Furthermore, airflow is dynamic, which affects the nasal condensation area. Finally, the test instrument does not measure nasal pressure.

Care was also taken when using the GM, such as making sure that subjects were in a correct seated position, that their head was orthostatic, and that placement of the metal plate was centralized and horizontal. This positioning is still subject to tilting errors.

It is known that nasal airway resistance - the third component that affects measurement variability - may change abruptly in response to various stimuli. For example, exercise and warm air decrease the resistance, while cold air, cigarette smoke, pain, pregnancy, and hypoventilation increase the resistance.^35^ Smokers, users of topical or systemic medication and pregnant women were excluded to control these variables. Furthermore, participants were asked to refrain from drinking alcoholic beverages and from taking medication; they also remained 30 minutes seated in the exam room - in which temperature and humidity were constant - for acclimatizing purposes to minimize the abovementioned variables. Daily measurements were always done at about the same hour. During the measurements, participants were asked to breathe calmly, with eyes and mouth closed. The subject's voluntary breathing, however, was not controlled. Expiration with increased or decreased effort may be perceived, which may be corrected; but there is no absolute control over inspiration and expiration. The association between these factors may have decidedly affected the variability of results.

Median unilateral variation coefficients (VCs) were below 15%; total VCs were below 12% for different time intervals. Two findings stand out in our data: total coefficients were lower than unilateral coefficients, and there was little variability of results when comparing different time intervals.

An explanation for the first aspect appears simple. There are periodic alterations of unilateral nasal patency as a function of the nasal cycle; one side increases and the other decreases. This cycle results in significant variability in unilateral nasal flow measurements.^37^ Total airflow, however, tends to remain roughly constant,^38^, ^39^, ^40^ which explains lower VCs.

As to the second aspect, it might be assumed that there was adequate control of factors that might have affected the time variability of the technique. On the other hand, these results differ from those found in other studies;^14^^,^^16^, ^17^^,^^40^ would the test be sensitive enough to detect such differences? In this case, test accuracy, as well as reproducibility, should be taken into account, that is, the ability of the test to represent the essence of a situation or measured quantity.^36^ Although there is no universally accepted gold standard for the accuracy of nasal patency measurements, comparative studies with other evaluation methods of nasal function would be interesting.

Daele and De Vos^11^ introduced artificial obstacles in different sites of the nasal fossae of three normal subjects (the internal ostium, the median portion and the posterior portion) to investigate the correlation between active and passive anterior rhinomanometry with the GM. The correlation between methods was best when the obstacle was placed in the internal ostium.

Fisher et al.^6^ used acoustic rhinometry and rhinohygrometry (plate described by Gertner) to observe the “nasal cycle,” in 15 children aged from 3 to 10 years and no evidence of nasal disease. There was poor agreement between both methods (47% Kappa = −0.17).

Given the lack of studies showing the VCs of the nasal condensation area, we chose to discuss our results with data collected by using other nasal respiratory function evaluation techniques in a similar population and in baseline conditions.

Given the high nasal resistance variability in rhinomanometry (which is a dynamic technique, like the GM), we highlighted studies that had similar,^7^^,^^16^ higher^17^^,^^37^^,^^40^ and lower^41^ VCs compared to our findings. The variability difference is more significant when a comparison is made with minimal acoustic rhinometry cross-sectional area VCs (static technique), which are lower.^7^^,^^14^, ^15^ Such findings agree with those described by Roithmann et al.,^14^ whereby static results are less reproducible than dynamic results.

The GM fulfils many of the criteria presented by Pallanch et al.^12^ as desirable for nasal airway assessments. The mirror is easy to use and requires simple training; it causes no discomfort for patients and does not affect nasal anatomy or airflow, as it is placed externally under the patient's nostrils. Furthermore, it is an inexpensive method. Reproducibility of the technique used in the current study was similar or lower than that found in other nasal flow tests, such as rhinomanometry. Its use may be standardized by paying attention to body and head posture, tilting of the plate, care with handling, the manner by which condensation is drawn, guidance of patients and calculation of the nasal condensation area. However, like other nasal function evaluation techniques, this method correlates poorly with subjective measurements of nasal patency.

There is only one article^9^ presenting data on normal nasal patency values as represented by the nasal condensation area; there are also few articles^10^ presenting results after therapeutic interventions. Furthermore, as mentioned above, the accuracy of the technique requires further studies for comparison with the gold standard, composed of the clinical history and physical examination of patients, as well as better established objective tests such as rhinomanometry and acoustic rhinometry.

Based on these comments, various aspects of the applicability of the GM in phonoaudiological and otorhinolaryngological clinical practice should be revised.

Flow asymmetry may be explained by the periodic alternation of physiological congestion and decongestion in each side of the nose. The nasal cycle, which has been observed in 72% to 80% of individuals, lasts on average about 2.9 hours.^6^ Continuing asymmetry of the condensation area and variability of measurements and sides of the nose in allergic cases^2^ cannot always be explained as probable mechanical obstruction to the passage of air,^2^ as has at times been suggested. Use of the total area is recommended as a parameter for recording the nasal patency.

In the current study, median VCs were about 11% and 14%, reaching 31% (total area) and 53% (unilateral area) in minute-by-minute interval measurements under controlled environmental and intrinsic conditions, and no therapy. These values should be used for interpreting the results of systematic measurements, as proposed by some authors.^2^ Additionally, measurements at the beginning of sessions do not taken into account environmental temperature and humidity differences, and physical activity or stress, all of which may affect the result variability.

Thus, when applying the GM for assessing nasal patency, we suggest using the mean of three to five recordings, carefully observing well known aspects in phonoaudiology, such as body and head posture, slow breathing and keeping the eyes and mouth closed; the aim is to avoid possible gratification behaviors by increasing or decreasing the breathing intensity, which children may exhibit.^6^

Interpretation of the nasal condensation area should always be done together with the clinical history (the patient's symptoms) and other data collected by examination of the oral sensory-motor system. It is also essential that cases with probable respiratory alterations be referred to otorhinolaryngologists for a specialized evaluation.

## CONCLUSION

The results revealed no correlation between subjective perception and nasal condensation area as measured by the GM. The median GM minute-by-minute unilateral variability was less than 14%. The median variability for total values (right and left) was less than 11%. There was no variability difference in the measurements of nasal condensation on the GM among different test retest time periods. Thus, professionals using the GM should be aware of the variability of this technique in the test and retest, as well as its lack of correlation with the perception of nasal patency.
